# Supplementary material for: A New Zebrafish Model to Measure Neuronal α-Synuclein Clearance In Vivo
Source: Genes (Basel). 2022 May 12;13(5):868. doi: 10.3390/genes13050868 (PMC9141618; doi:10.3390/genes13050868)
Supplement: Supplementary file 1 [file genes-13-00868-s001.zip › Supplementary Figure legends modified for proofreading.pdf]

Figure S1. Generation of transgenic Dendra- α-SYN zebrafish.

(**A**) Schematic representation of the responder constructs comprising the cDNA encoding human wildtype or mutant A53T α-SYN fused to the coding sequence for Dendra2 (Dendra-α-SYN) downstream of UAS. The construct also includes a selection marker in the reverse orientation comprising the *myl7* promoter driving expression of EGFP. Transgenes were flanked by Tol2 sites to facilitate random integration of the construct into the zebrafish genome. The UAS-GAL4 system was used to induce gene expression in a tissue-specific manner. **(B-C)** Quantification of the relative expression levels of GAL4 (B) and Dendra (C) by qPCR in individual clutches from 3 different founders of Dendra-α-SYN-wt (in blue) and A53T fish (in green) at 1, 2 and 3 d.p.f. The mRNA levels show variability in the expression of GAL4 and Dendra among different founders but a similar tendency for GAL4 to increase and the reduction of Dendra expression over time. Red squares represent selected founders with comparable expression levels used to create the stable lines. **(D-E)** Western blot for mouse anti-α-SYN and rabbit anti-Dendra in whole fish lysates from Dendra-α-SYN-wt and A53T mutant fish with neuronal expression at 1, 2 and 3 d.p.f. (D), quantified in graph in (E). The ratios between the antibodies detecting Dendra-α-SYN fusion protein and tubulin as the loading control were maintained (Data represented as mean ± s.d.; N=2 independent clutches per age; *ns* $p>0.05$ when comparing α-SYN/Tub or Dendra/Tub ratios at the same age). (**F**) Western blot for α-SYN and pSer129 (black arrowhead) showing detection of the phosphorylated form of α-SYN only in Dendra-α-SYN-A53T fish at 3 d.p.f. (white arrowhead points to an unspecific band detected when blotting with pSer129 also present in transgene-negative fish). (**G**) Representative brightfield and Dendra-fluorescence images of larvae with ubiquitous expression of Dendra-α-SYN-wt and A53T at 3 d.p.f. Scale bar

represents 1 mm. **(H)** Representative images of pools of 3 d.p.f. larvae with ubiquitous expression of Dendra-α-SYN-wt and A53T showing the absence of any morphological abnormality. Scale bar represents 1 mm.

Figure S2. Extended characterisation of neuronal pathology.

(**A**) Time course quantification of TUNEL labelling on five transverse cryosections of a Dendra-α-SYN-wt (WT) fish and a negative sibling (Neg.) used to identify the best age to see differences in cell death, compared to non-expressors. These results suggest that fish with pan-neuronal expression of the transgene showed a larger number of apoptotic cells at 2 d.p.f. than transgene-negative siblings. This time point was used to compare the effect of Dendra-α-SYN-wt and A53T in main Fig. 2A&B. Data represents mean ± SEM (N=5 sections from same fish) (**B**) Representative confocal pictures of sections across the spinal cord used for antibody immunostaining with GFAP showing similar staining patterns in Dendra-α-SYN-wt and A53T, compared to transgene-negative siblings at 3 d.p.f. Scale bar represents 50 µm. (**C**) **i.** Schematic representation of the 5 axons used to quantify the abnormalities in the straightness, branching (ramification to innervate medial myotome highlighted by yellow arrowhead in i&ii) and length (innervation of ventral myotome highlighted with blue arrowhead in i&ii); **ii.** Representative z-stack projection from confocal images of normal motor neuronal axons labeled with antibody staining for alpha-acetylated tubulin in wholemount 3 d.p.f. Dendra-α-SYN-A53T (quantification presented in Fig. 2C). Scale bar represents 100 µm. (**D**) Quantification of the branching (ramifications), parallel axons (straightness) and length (axons reaching the ventral myotome) of motor neurons shown as mean (red line) ± s.d. (N=5 fish/group; ns $p>0.05$). A total of 5 axons per fish were quantified. No defects were found in the morphology of motor neuron axons in positive expressors (+)

when compared to negative siblings (-). (**E**) Quantification of escape response to tail touch in Dendra-α-SYN-wt and A53T and their respective negative siblings at 3 d.p.f. (3 independent experiments in triplicate shown as mean ± s.d, N=20/group; ns p>0.05 vs. negative siblings; ns p>0.05 ANOVA). (**F**) Quantification of the relative mRNA levels of Dendra by qPCR in fin clips collected from individual fish with ubiquitous expression of Dendra-α-SYN-wt (wt) and A53T fish at 16 weeks old. Data represented as mean (red line) ± s.d. (N=9 fish per group; (*ns p>0.05* wt *vs.* A53T).

Figure S3. Dendra-α-SYN-wt and A53T expression results in aggregate formation.

(**A**) Z-stack projections from confocal images of the spinal cord in living Dendra-α-SYN-wt and A53T fish with pan-neuronal expression. Pictures show the abundance of bright puncta along the axons in the spinal cord in both transgenic fish. Scale bar represents 100 µm. (**B**) Representative confocal images of the spinal cord in transgenic lines with mosaic expression of Dendra-α-SYN (top), Dendra-tau-A152T (middle) or Dendra alone. Puncta can be observed in axons from Dendra-α-SYN transgenic fish (hollow arrowheads), whereas Dendra-tau-A152T nor Dendra only fish show homogenous fluorescent signal along the axons (white arrowheads). (**C**) Confocal images of different cell types expressing Dendra-α-SYN from crosses with *EIf1α*:GAL4-VP16 showing the lack of puncta at 3 d.p.f. Despite the bright fluorescent signal found in interneurons, only few puncta were observed. Scale bar represents 50 µm. **(D)** Representative images of transverse cryosections across the head of 3 d.p.f. embryos fixed in PFA used for the quantification of brain puncta represented in main Fig. 3B. Panel i. offers a schematic representation of what can be seen in the images of Dendra fluorescent signal from Dendra-α-SYN-wt (ii-iv) and Dendra-α-SYN-A53T (v-vii). Red dots in panels iii. and vi. correspond to puncta quantified in that picture. Panels iv. and

vii. represents magnified images to facilitate the visualisation of the puncta. Scale bar represents 100 µm.

Figure S4. Dendra-positive puncta correspond to aggregate-like structures of Dendra-α-SYN.

(A-B) Confocal images from immunofluorescence staining for acetylated tubulin with intracellular localisation (A) and membrane-bound synaptotagmin-2 (znp-1) (B) to investigate whether Dendra-α-SYN puncta formation occurs as a consequence of changes in axonal morphology and integrity. Images show DAPI, Dendra and neuronal antibody signals in longitudinal cryosections across the hindbrains of 3 d.p.f. larvae with neuronal Dendra-α-SYN-A53T expression and transgene-negative siblings. No differences were seen in the pattern of red signal compared to non-expressors. Scale bar represents 150 µm. **(C)** Representative images of Dendra signal in cryosections across the head of 1, 2 and 3 d.p.f. fish with pan-neuronal expression of Dendra-α-SYN-A53T to show the increase in dendra-positive puncta over time. Scale bar represents 100 µm.

Figure S5. Autophagy inhibition reduces the clearance kinetics of Dendra-α-SYN-wt.

(A) Graph represents the differences in the clearance rate of Dendra- α-SYN-wt in the presence or absence of ammonium chloride (NH_4Cl) to block lysosomal acidification and autophagic flux. NH_4Cl delays the clearance of Dendra-α-SYN-wt (N=minimum 30 neurons per group; ****$p<0.0001$ vs. untreated α-SYN-wt).
