# Supplementary material for: A New Zebrafish Model to Measure Neuronal α-Synuclein Clearance In Vivo
Source: Genes (Basel). 2022 May 12;13(5):868. doi: 10.3390/genes13050868 (PMC9141618; doi:10.3390/genes13050868)

Figure S1. Generation of transgenic Dendra-tau zebrafish.

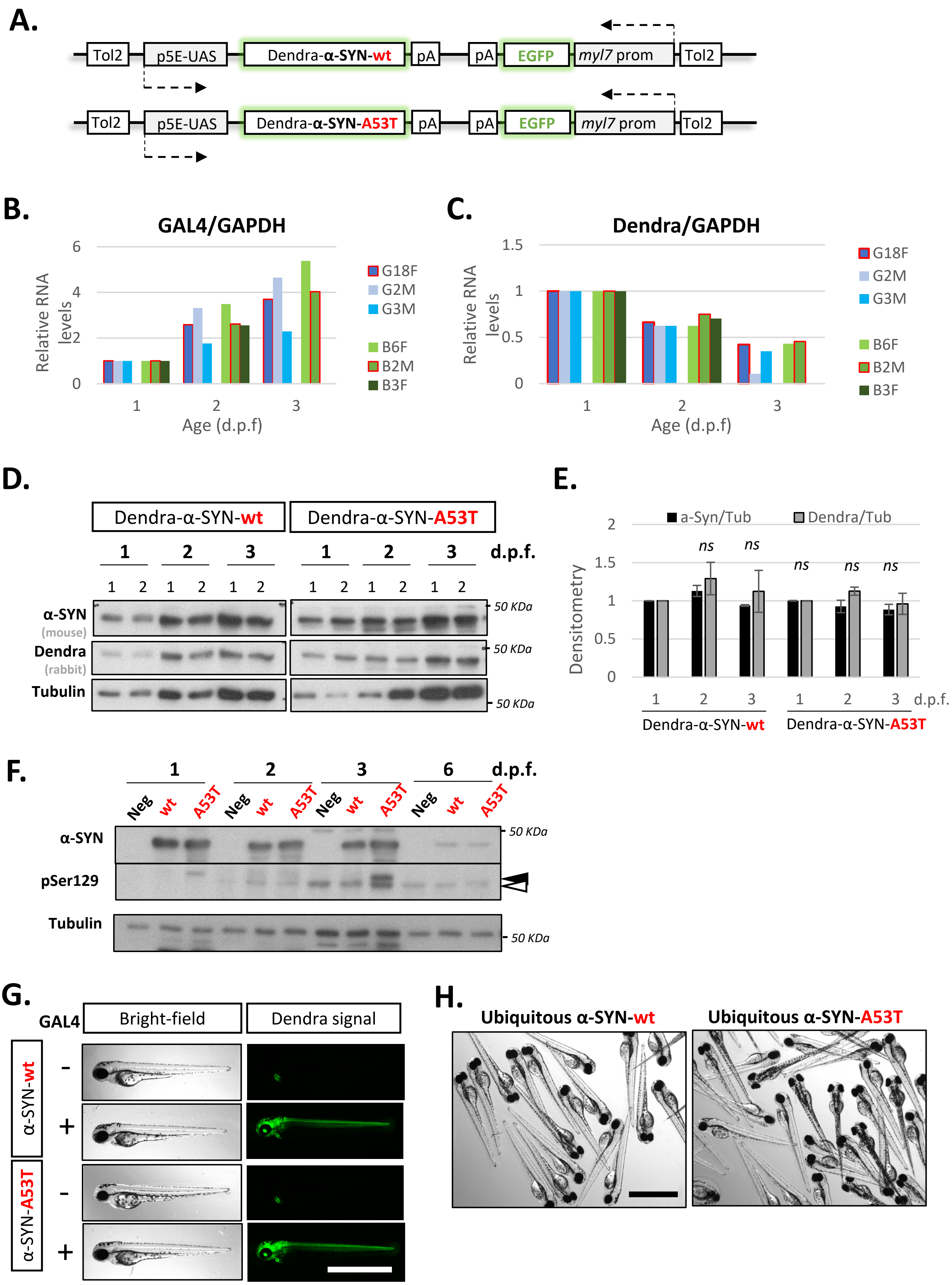

Figure S2. Extended characterisation of neuronal pathology.

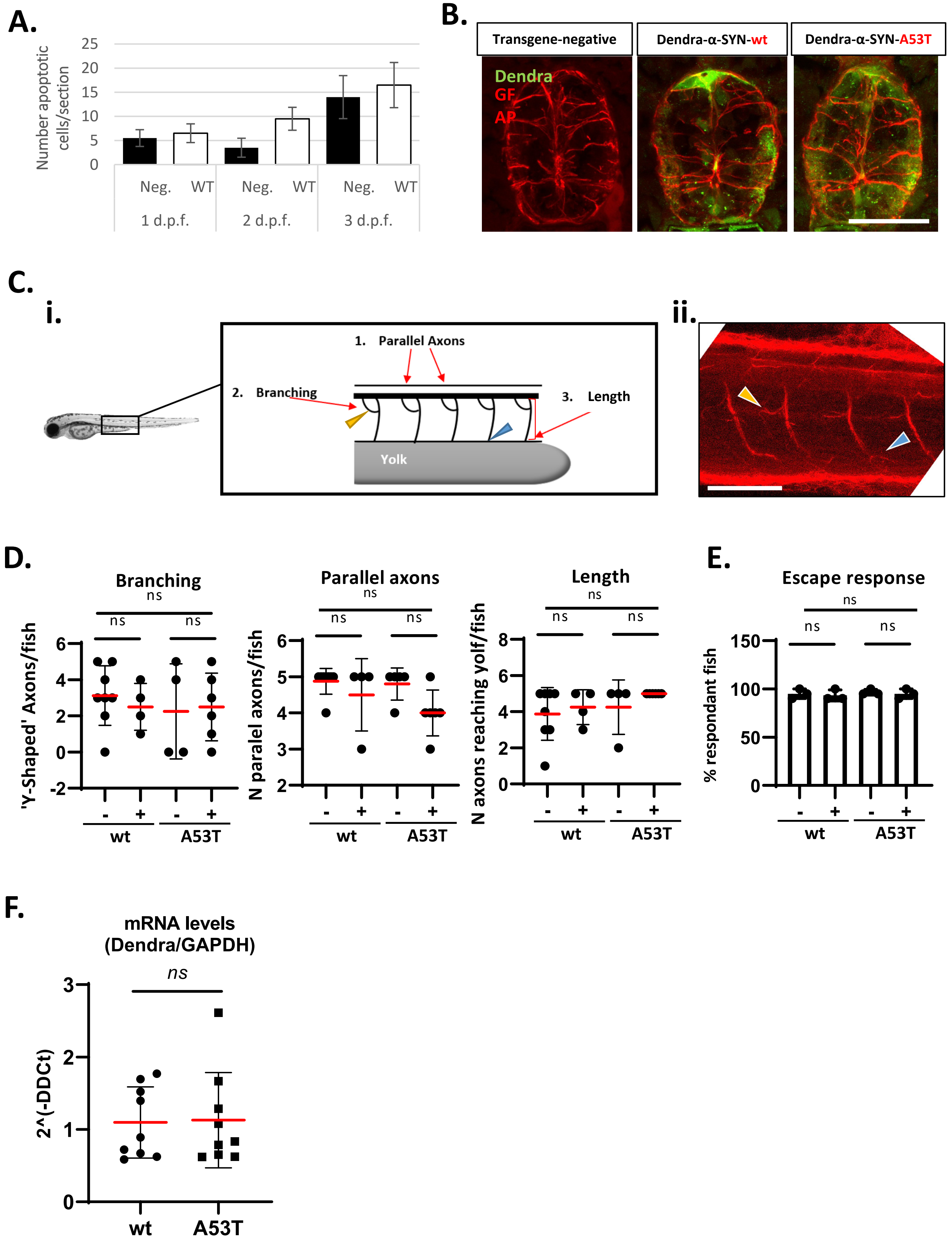

Figure S3: Dendra-α-SYN-wt and A53T expression results in aggregate formation.

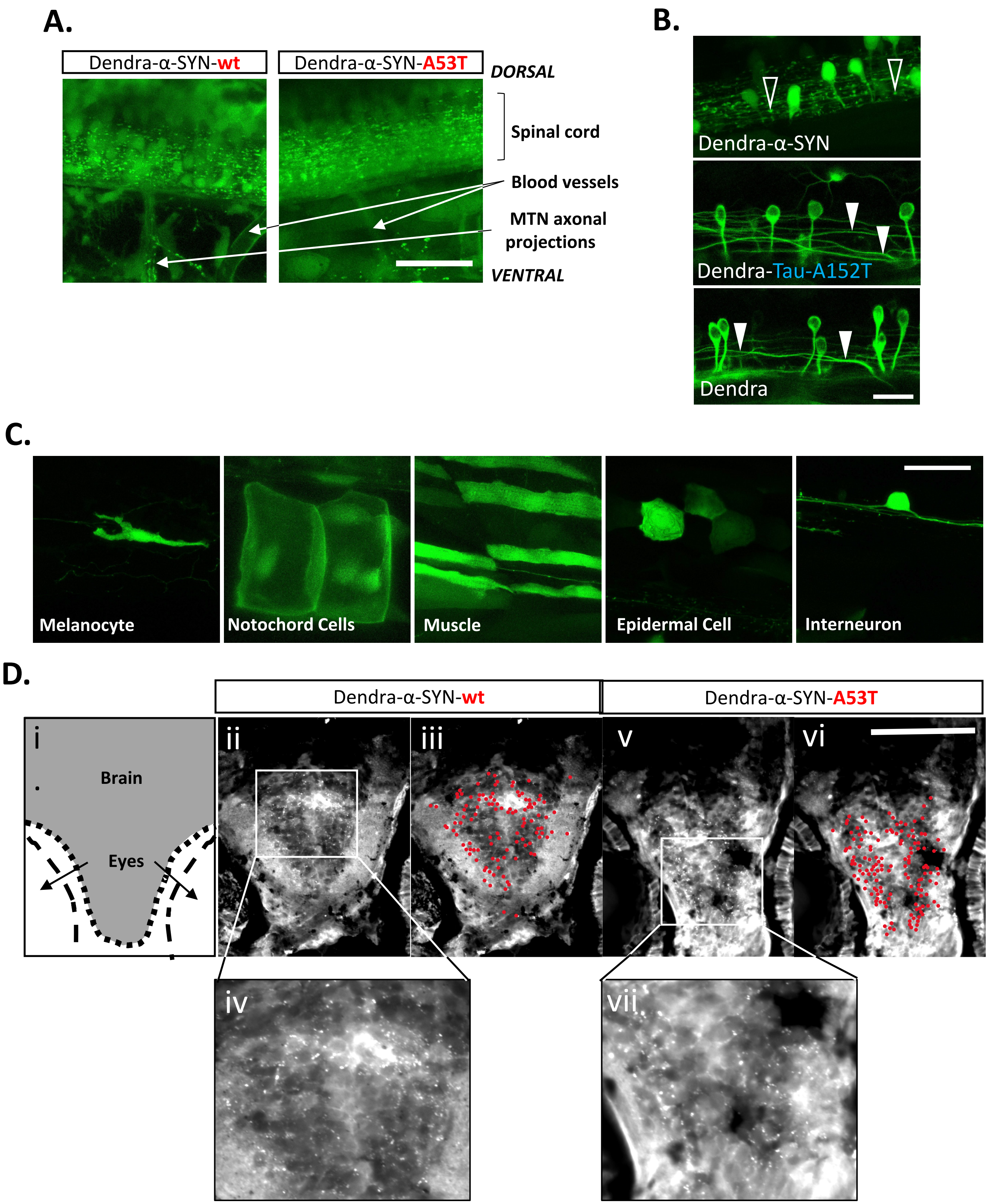

Figure S4. Dendra-positive puncta correspond to aggregate-like structures of Dendra-α-SYN.

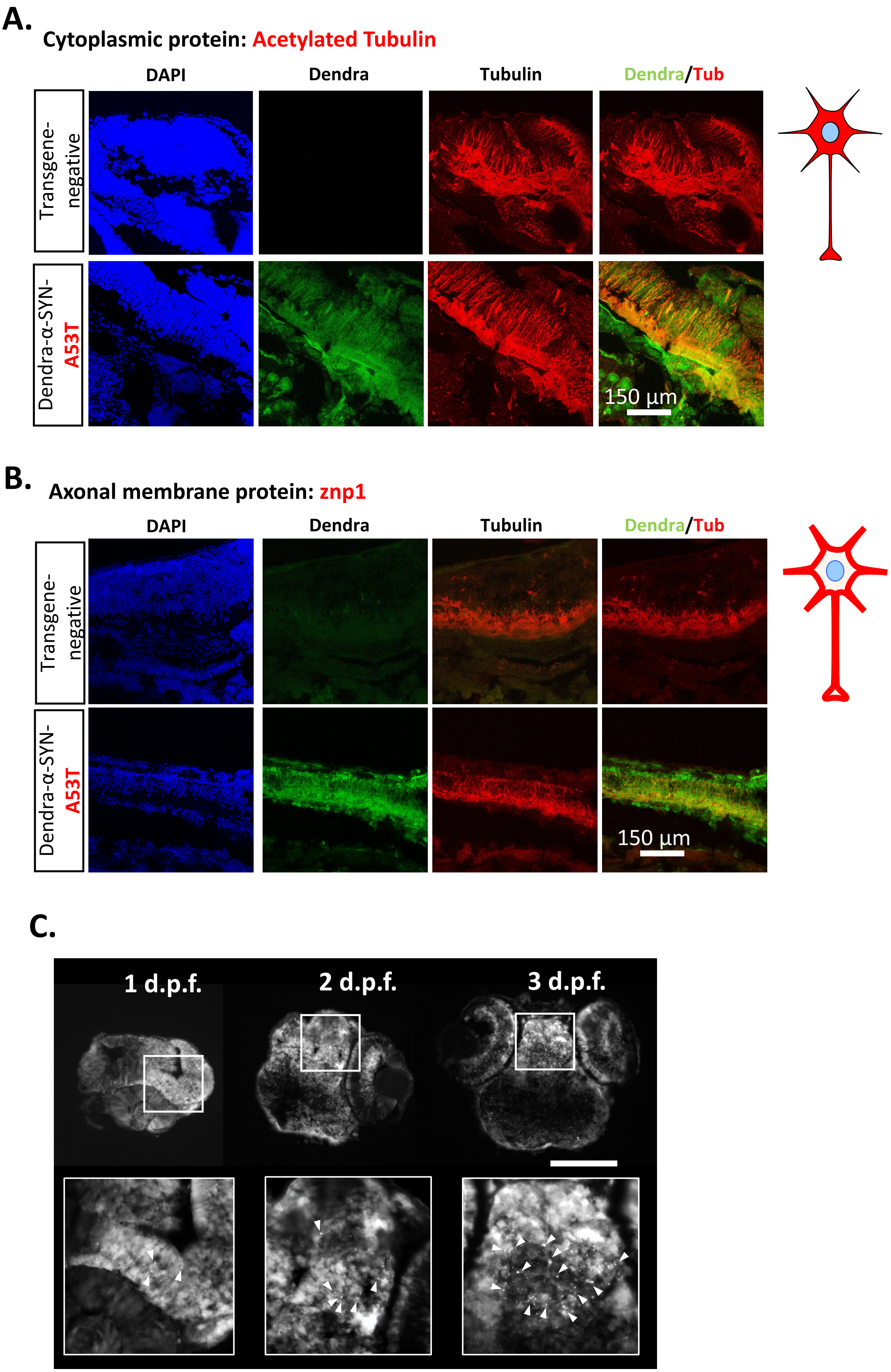

Figure S5. Autophagy inhibition reduces the clearance kinetics of Dendra-α-SYN-wt.

A.

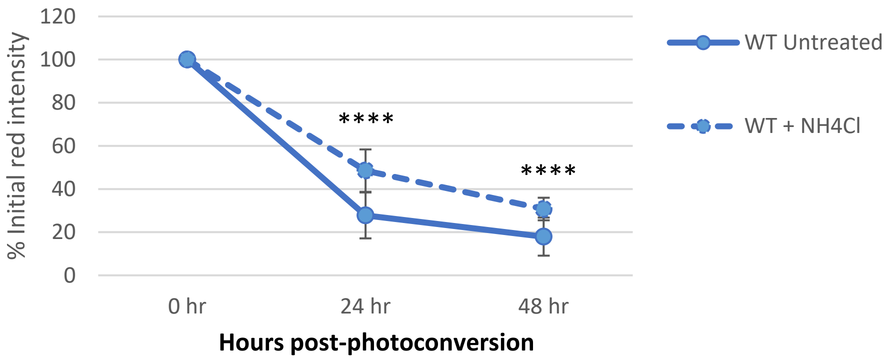

Supplement: Supplementary file 1 [file genes-13-00868-s001.zip › Supplementary Figures including new data for revision.pdf]
